# Co-production of biohydrogen and biomethane utilizing halophytic biomass *Atriplexcrassifolia* by two-stage anaerobic fermentation process

**DOI:** 10.3389/fchem.2023.1233494

**Published:** 2023-07-07

**Authors:** Ali Nawaz, Farheen Aamir, Rong Huang, Ikram ul Haq, Fangyu Wu, Marium Munir, Rida Chaudhary, Ayesha Rafique, Kankan Jiang

**Affiliations:** ^1^ School of Basic Medical Sciences and Forensic Medicine, Hangzhou Medical College, Hangzhou, China; ^2^ Institute of Industrial Biotechnology, Government College University, Lahore, Pakistan; ^3^ Food and Biotechnology Research Center, Pakistan Council of Scientific and Industrial Research, Lahore, Pakistan; ^4^ Institute of Molecular Biology and Biotechnology, The University of Lahore, Lahore, Pakistan

**Keywords:** anaerobic digestion, dark fermentation, *Atriplexcrassifolia*, acidogenesis, methanogenesis, biohydrogen, biomethane

## Abstract

The excessive use of fossil has resulted in the drastic exhaustion of natural energy sources, leading to environmental challenges and energy crises. Owing to rising energy demand there is a dire need to shift towards renewable energies from lignocellulosic biomass. The present study assessed the co-production of biohydrogen (H_2_) and biomethane (CH_4_) by utilizing a less explored halophyte *Atriplexcrassifolia.* Various reaction parameters were evaluated for their effect on biohydrogen and biomethane production in batch experiments. One parameter at a time experimental strategy was chosen for production optimization. Hydrogen and methane yields along with their production rates were assessed at different incubation times, temperatures, pH, substrate concentrations, and inoculum sizes in acidogenesis and methanogenesis stages, respectively. In the first stage, maximum cumulative hydrogen production of 66 ± 0.02 mL, with hydrogen yield of 13.2 ± 0.03 mL/g, and hydrogen production rate (HPR) of 1.37 ± 0.05 mL/h was attained when the reaction mixture (5 g *Atriplexcrassifolia* and 10 mL pretreated sewage sludge) was processed at 37°C and pH 5.5 after 48 h of incubation. While in the second stage, maximum cumulative methane production, i.e., 343 ± 0.12 mL, methane yield (MY) of 8.5 ± 0.07 mL/mL, and methane production rate (MPR) of 0.8 ± 0.05 mL/h was achieved after 18 days of incubation of reaction mixture (40 mL of hydrogenic slurry with 80 mL inoculum) at 45°C and pH 8. Furthermore, a 51% and 24% rise in biohydrogen and biomethane production respectively were recorded when the gases were produced at these optimized reaction conditions. The results ensure halophyte *Atriplexcrassifolia* as an imperative renewable energy resource and proposed that effective optimization of the process further increased the coproduction of biohydrogen and biomethane.

## 1 Introduction

Over the past few decades, excessive exploitation of fossil fuels has exacerbated the depletion of natural resources, leading to environmental degradation, global warming, and climate variability brought on by overpopulation and rapid urbanization. According to International Energy Agency, 2020, 88% of the world’s energy demand is fulfilled by fossil fuels (Aghbashlo et al., 2018; Hosseinpour et al., 2018), resulting significant increase in the atmospheric concentration of greenhouse gases (GHG), with global CO_2_ emissions reaching 33.1 Gt (British Petrol Energy Outlook, 2019). These worldwide challenges prompted renewed interest in renewable energy sources (Banu et al., 2020; Sharmila, et al., 2020). Biofuels are significant existing environment-friendly energy assets around the globe. According to ongoing improvements in EU policies, 32% of the world’s energy demand will be fulfilled in 2030 by renewable resources (EU. Red II Directive).

Lignocellulosic biomass is ample and inexhaustible feedstock for bio-energy with global accessibility of around 220 billion tons per annum, attributing to 10% of worldwide energy demand (Radhakrishnan et al., 2020). Conventional methods utilized first-generation food crops (starchy grain and maize) as feedstock. Researchers are now focusing on exploiting lignocellulosic biomass such as halophytes to produce second-generation biofuels in an attempt to overcome the conflict between food and biofuel in developing countries (Sharma et al., 2016). With yields comparable to other energy crops and the additional advantage of utilizing saline soils, halophytic plants have enormous potential to produce biofuels. Halophyte *Atriplexcrassifolia* (saltbush) belongs to the Amaranthaceae family. Among other species, it can be found all over the world in a wide range of environments, including preserved alkaline and salt deserts, tropic soil, semi-natural meadows, irrigated agricultural fields, and coastal areas (Hasanuzzaman et al., 2019). Halophyte-based biofuels promise to strengthen the global economy while simultaneously assuring financial stability (Prasad and Ingle, 2019). This study ensures halophyte *Atriplex crassifolia* as an imperative renewable energy resource.

Among various biofuels acquainted to date, hydrogen (H_2_) and methane (CH_4_) are expected to play a significant role in the green economy (Aghbashlo et al., 2019). Biohydrogen is accounted as a potentially viable alternative to fossil fuels, due to its high energy density (142 kJ/mol), being 2.75 times greater than other fuels (Abdullah et al., 2020). H_2_ burning merely emits water vapors with no hazardous gases into the atmosphere (Zhang et al., 2020). Methane comprises the majority of biogas because of its greater energy density (Srivastava et al., 2020). It has all the advantages of petroleum, including a robust network for transportation, trading, and supply (Rasapoor et al., 2020; Fu et al., 2021). To compete with present-day fuel demand, appropriate methods are required to produce hydrogen and methane. Despite the advantages of single-stage dark fermentation or anaerobic digestion (AD) processes, novel techniques must employ for global biogas production (Rasapoor et al., 2020; Paritosh et al., 2021). Recent research has led to the development of two-stage anaerobic digestion process, which divides into two different phases (acidogenesis and methanogenesis) (Thungklin et al., 2018). The co-production of H_2_ and CH_4_ in the two-stage AD process is thought to be a significant pathway to increase energy recovery by 90% because effluent at the end of dark fermentation (acidogenesis) is rich in organic matter. It is mostly due to the simple operational process, enhanced production rate, improved COD reduction, and wide range of feedstock availability (Rabii et al., 2019).

Incubation time, temperature, pH, substrate concentration utilized, and inoculum size are significant environmental and operational factors in a two-stage process. Incubation time has been reported to perform a significant part in increasing the hydrogen and methane yields (Mohan et al., 2007; Lin P. J. et al., 2011) and might significantly influence the composition of produced volatile fatty acids in effluent (Liu et al., 2008). Production rates of both CH_4_ and H_2_ increase within a certain time range, but when the optimum time limit exceeds, productivity decreases as incubation time increases. Specific factors affecting incubation time include substrate used and biodegradability (Bakonyi et al., 2018). According to some reports, acidogenic bacteria’s ability to produce hydrogen can increase with temperature within a specific range but can also be inhibited by excessive heat (Wang and Wan, 2009). The optimum temperature range for the anaerobic digestion process depends on micro-flora. pH affects the activity of hydrogenase and other metabolic processes, therefore choosing the optimum pH is also essential for increasing H_2_ and CH_4_ production. While Lee et al. (2002) claimed that the maximum H_2_ yield rate was attained at an initial pH of 9, Fan et al. (2004) and Ginkel et al. (2001) stated the highest cumulative H_2_ production at pH 5.5. These contradictory results indicated to be the consequence of a reduction in buffering capacity, which stopped pH from decreasing. From a practical perspective, it's crucial to investigate how initial pH affects hydrogen and methane production when there is no pH control during anaerobic fermentation.

The majority of research on optimizing the AD process focuses on the substrate and inoculum concentration. Reduced substrate concentration operates as a limiting factor and inhibits the synthesis of biogas, however, if it exceeds its optimum limit, all pre-available enzymes form enzyme-substrate complexes and may not be able to bind with the residual substrate. Few studies have specifically examined the role of inoculum size. It is well acknowledged that inoculum is crucial in a two-stage anaerobic digestion process to preserve the system’s stability and hasten the initiation of digestion (De Vrieze et al., 2015). Quintero et al. (2012) claim that the production of biogas, the pace at which organic matter breaks down, the length of the lag phase, and the stability and ease of scaling of the AD can all be improved by the use of the appropriate inoculum. The substrate-to-inoculum (S:I) ratio affects AD because a suitable S:I can regulate the inoculum’s microbial population and make the hydrolysis step easier (Li et al., 2018). The effects of substrate concentration and inoculum size on the two-stage anaerobic digestion process are still not fully understood.

Biohydrogen and biomethane have drawn worldwide consideration. Both are effective alternatives to fossil fuels due to their favorable environmental effects, simplicity of usage in industry, and potential to reduce developing nations’ reliance on fossil fuels. As mentioned previously, this research project will be the first to assess the co-production of biohydrogen and biomethane in a two-stage anaerobic digestion process utilizing unexplored halophyte *Atriplexcrassifolia*. Moreover, the present study was also carried out to investigate the effects of hydraulic retention time, temperature, pH, substrate concentration, and inoculum size on efficient biohydrogen and biomethane production from pretreated *Atriplexcrassifolia* using sewage sludge microflora.

## 2 Materials and methods

### 2.1 Substrate and pretreatment

*Atriplexcrassifolia,* a halophyte, was gathered from the fields of GCUniversity Lahore, Kala Shah Kaku (KSK) campus, Pakistan. This air-dried lignocellulosic biomass was crushed into a fine powder (1.5 mm) and passed through a 16-mesh sieve before being employed as a substrate. Alkali pretreatment of the substrate was done utilizing 3% sodium hydroxide (NaOH) reagent, heating at 121°C for 60 min Table 1, summarizes the detailed composition and characterization of *Atriplexcrassifolia*.

**TABLE 1:** Characterization of pretreated and untreated *Atriplexcrassifolia* used for two stage anaerobic digestion (AD).

Characteristics	Untreated *Atriplexcrassifolia*	Pretreated *Atriplexcrassifolia*
Lignin (%)	19.2% ± 0.02%	6.9% ± 0.02%
Cellulose (%)	37% ± 0.04%	62% ± 0.07%
Hemicellulose (%)	21% ± 0.02%	12.3% ± 0.06%
Delignification (%)	__	64% ± 0.01%
TS (%)	95.6% ± 0.12%	98.5% ± 0.03%
VS. (%)	94.8% ± 0.03%	95.3% ± 0.07%

### 2.2 Seed inoculum

The inoculum (municipal sewage sludge) was obtained from the Hydrology Directorate and WASA laboratory, LDA, Lahore. For the first (acidogenesis) stage, the sewage sludge was subjected to the heat shock method according to Yang et al. (2019). This procedure involved heating the sludge for 15 min at 100°C. Acidogenic and methanogenic vegetative cells were destroyed by the heat shock method, whereas endospore-producing acidogenic cells remained intact. The characteristics of inoculum were; pH 9.5, total solid concentration (TS) of 13%, and volatile solid concentration (VS.) of 46%.

### 2.3 Fabrication

#### 2.3.1 Nitrogen purging

To assemble a nitrogen purging apparatus, a 5 mL syringe was utilized and cut in half, leaving 3 mL of space from the plunger side. To the plunger side of the syringe, a balloon was attached and securely sealed with para-film. Additionally, a 16 gauge LP needle was connected to the syringe, enabling the introduction of nitrogen gas for the purging process.

#### 2.3.2 Water displacement assembly

**FIGURE 1:**
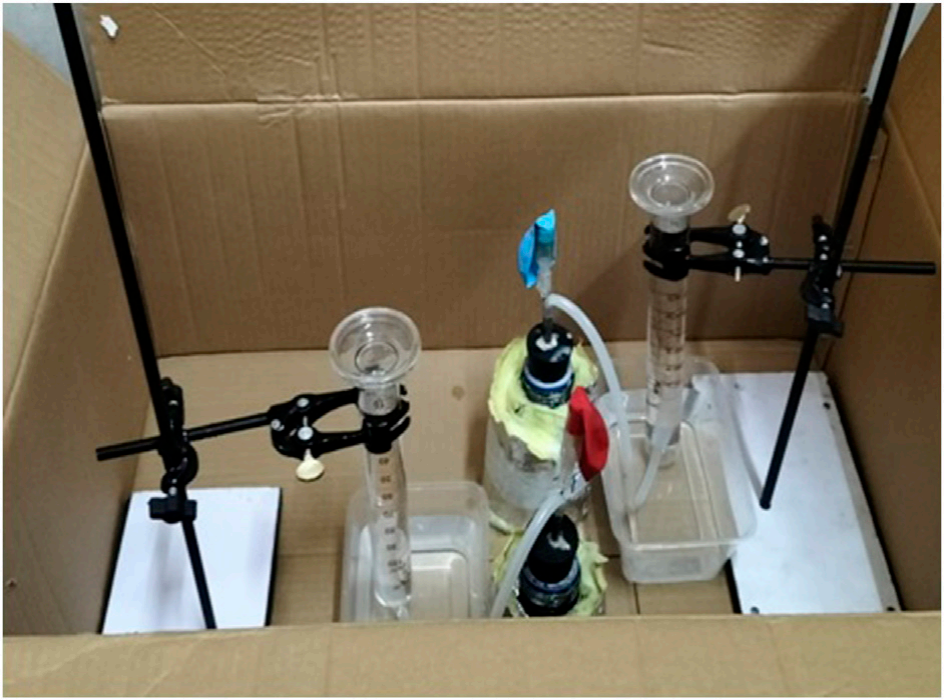
Water displacement assembly for biogas production.

Water displacement apparatus was assembled using various components. The setup included a 250 mL reagent bottle with a rubber cork, through which steel pipes were inserted. The steel pipe was then connected to a plastic pipe. An inverted measuring cylinder was positioned inside a plastic container, supported by a retort stand, with a distance of 1 cm–2 cm from the base. The plastic pipe was inserted through the base of the container, entering the measuring cylinder to facilitate the displacement of water (Figure 1).

### 2.4 Two stage anaerobic digestion setup

The two stages of the experiment involved dark fermentative hydrogen production (acidogenesis) and anaerobic digestion methane production (methanogenesis). Dark fermentative biohydrogen production experiments were performed in 250 mL reagent bottles with a working capacity of 200 mL. Each fermentative reagent bottle contained 5 g VS. *Atriplexcrassifolia,* 48 mL of hydrogen fermentation media (g/L) i.e., CuCl_2_ (0.01 g), KH_2_PO_4_ (0.25 g), NH_4_Cl (0.5 g), MnCl_2_ (0.015 g), K_2_HPO_4_ (0.25 g), ZnCl_2_ (0.01 g), FeCl_3_ (0.02 g), CaCl_2_ (0.005 g) and MgCl_2_ (0.3 g) (Sekoai and Kana, 2013), 25 mL pretreated inoculum. Sodium bicarbonate (5 g/L) was added for buffering capacity. To provide anaerobic environment, nitrogen gas was injected into the headspace of each reagent bottle. All reagent bottles were manually shaken for 1 min after a few hours to provide agitation.

In the second stage (methanogenesis), under various fermentation conditions effluent (slurry) from the hydrogen production step was transferred to reagent bottles with a working volume of 200 mL. The substrate-to-inoculum ratio (1:2) was added to each reagent bottle. All bottles were once again sealed before being incubated to produce methane. Initial pH in both stages was maintained by adding 1N NaOH and 1 N HCL solution (Cheng et al., 2016). All of the experimental tests were performed in triplicates.

### 2.5 Effect of physiochemical parameters on biohydrogen and biomethane production

The physiochemical parameters optimized for H_2_ (biohydrogen) production were incubation time (1–7 days), pH (4.5–7), incubation temperature (25°C—40°C), substrate concentration (3g–7 g), and inoculum size (5 mL–25 mL). However, for biomethane production, the parameters were the same but the ranges varied such as incubation time (4–20 days), incubation temperature (35°C—55°C), pH (7–9), substrate/slurry concentration (10 mL–50 mL) and inoculum size (20 mL–100 mL).

### 2.6 Analytical technique

The lignocellulosic content of *Atriplexcrassifolia* was assessed using a technique developed by the NREL (National Renewable Energy Laboratory) (Sluiter et al., 2008), and VS. and TS were evaluated using standard techniques (APHA, 1998). The volume of biogas produced during the two-stage anaerobic digestion process was estimated using the water displacement method. A vessel filled with barrier solution was submerged in a reservoir. The amount of gas produced was equal to the volume of water that was displaced in the container (Yin et al., 2014). Based on the cumulative biohydrogen and biomethane production potential, maximum hydrogen yield (mL/g) and methane yield (mL/g) values were estimated for the batch experiment.

### 2.7 Statistical analysis

Following the completion of each experiment in triplicate, the results were statistically analyzed with SPSS version 16.00. (IBM Analytics, New York, United States). The data was represented graphically using MS Excel, and the Y-error bars in the result section’s figures showed the SD (standard deviation) amid triplicate tests, which varied considerably at *p* ≤ 0.05.

## 3 Results

### 3.1 Content analysis

Table 1 shows lignin, cellulose, and hemicellulose contents of untreated and pretreated *Atriplexcrassifolia.* Maximum delignification of 64% ± 0.07% and enhanced available cellulosic content of 62 ± 0.01%were observed by utilizing 3% NaOH (sodium hydroxide) reagent-mediated pretreatment.s.

### 3.2 Effect of incubation time on biohydrogen and biomethane production

**FIGURE 2:**
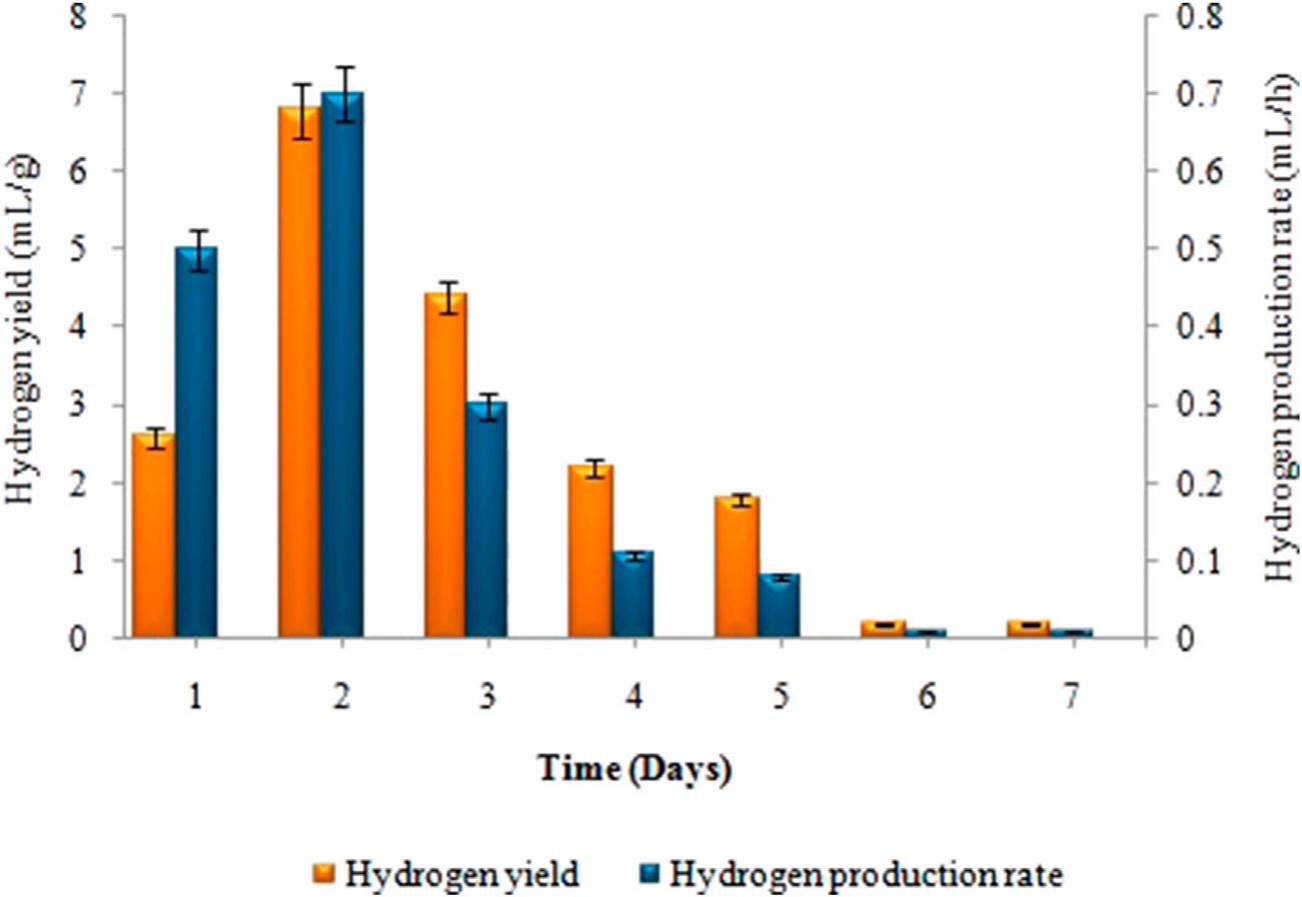
Effect of incubation time on H_2_ yield (mL/g) and H_2_ production rate (mL/h) in dark fermentation.

**FIGURE 3:**
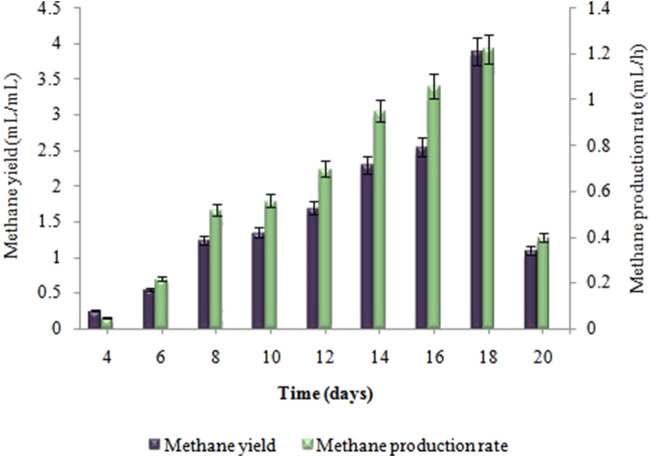
Effect of incubation time on MY (mL/mL) and MPR (mL/h) in anaerobic digestion.

Biogas compositional study revealed that produced biogas was entirely devoid of methane and only comprised hydrogen and carbon dioxide (i.e. 2.57: 1) respectively. The amount of biohydrogen produced over the first 24 h, i.e. 13 ± 0.02 mL increased gradually, reaching its peak after 2 days of incubation, i.e. 34 ± 0.05 mL. According to Figure 2, the maximal HY and HPR, i.e. 6.8 ± 0.03 mL/g and 0.7 ± 0.02 mL/h, respectively, were obtained after 48 h. A continued increase in incubation time led to a steady decline in the synthesis of biohydrogen. However, in the second stage the maximum cumulative methane production, i.e. 81 ± 0.07 mL, the highest MY, i.e. 3.95 ± 0.03 mL/mL, and the maximum MPR, i.e. 1.22 ± 0.04 mL/h were recorded on the 18th day of incubation (Figure 3). Biogas compositional study revealed that the produced biogas was majorly comprised of methane (i.e.,78%).

### 3.3 Effect of incubation temperature on biohydrogen and biomethane production

**FIGURE 4:**
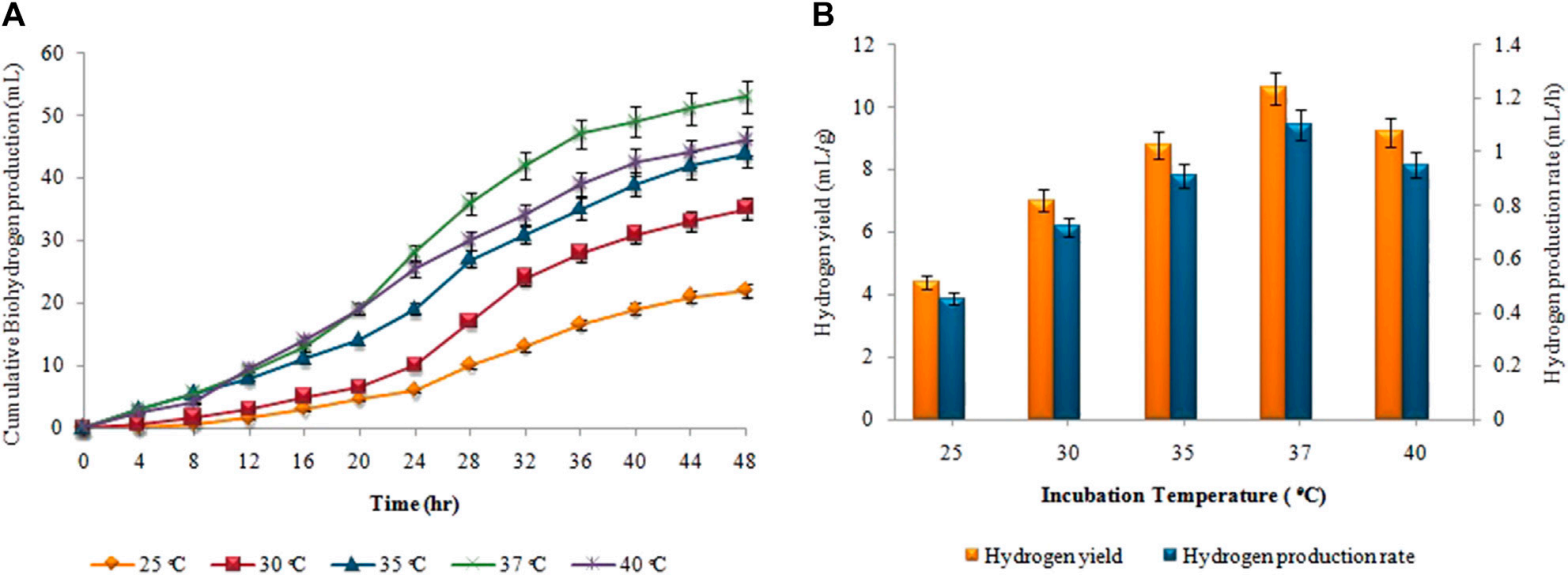
**(A)** Cumulative H_2_ production (mL), **(B)** H_2_yield rate (mL/g) and production rate (mL/h) at different incubation temperatures.

**FIGURE 5:**
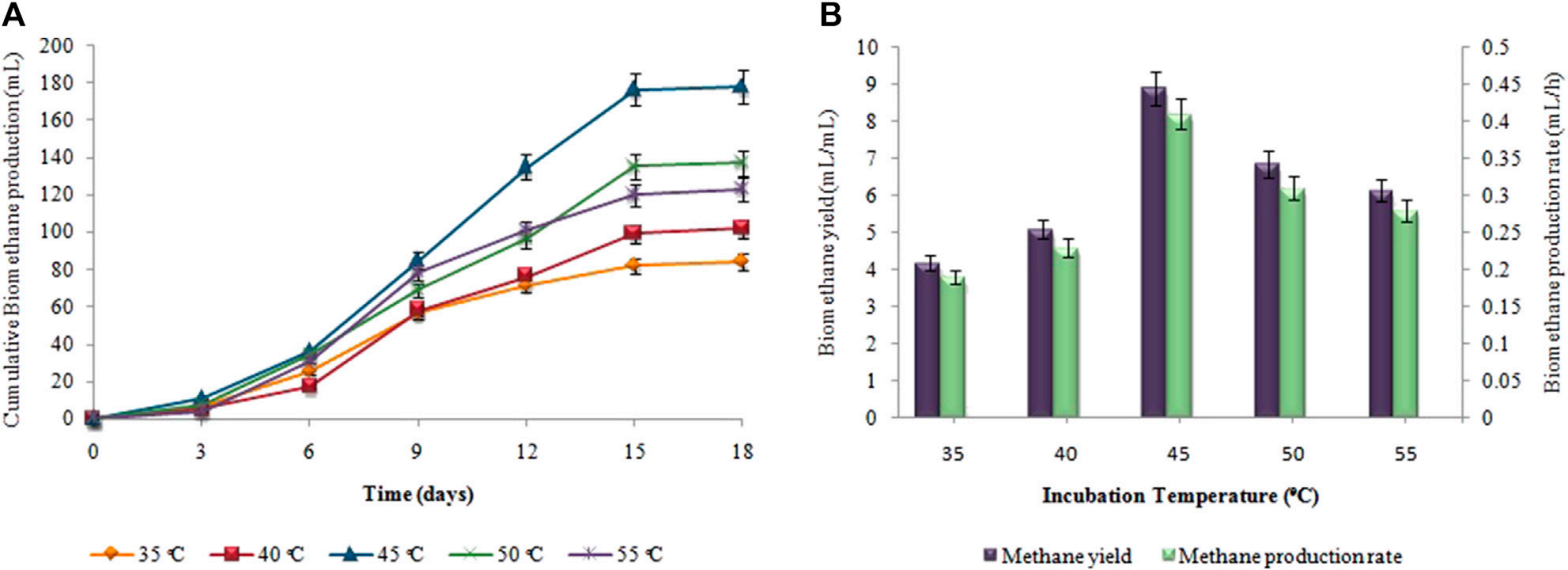
**(A)** Cumulative CH_4_ production (mL), **(B)** CH_4_ yield (mL/mL) and production rate (mL/h) at different incubation temperatures.

Figure 4, depicts the results of corresponding biohydrogen fermentation at varying temperatures. By comparing various temperature regimes (25°C–40°C), the optimum temperature for enhancing biohydrogen production was determined. According to Figure 4A, hydrogen production began progressively after a certain lag period and terminated within 48 h. When the temperature exceeded 30°C, there was a significant increase in the cumulative hydrogen production. Maximum cumulative biohydrogen generation was achieved at 37°C (53 ± 0.01 mL), followed by 40°C (46 ± 0.02 mL). The maximum HY and HPR, i.e. 10.5 ± 0.01 mL/g and 1.2 ± 0.03 mL/h, respectively, were also attained at 37°C (Figure 4B). However, in the second stage, as the temperature increased from 35°C to 40°C, the synthesis of biomethane gradually increased. The maximum cumulative biomethane production (178 ± 0.05 mL) was observed at 45°C (Figure 5A). While, the maximum MY of 8.9 ± 0.02 mL/mL and the highest MPR of 0.41 ± 0.03 mL/h were also achieved at 45°C, as shown in Figure 5B.

### 3.4 Effect of pH on biohydrogen and biomethane production

**FIGURE 6:**
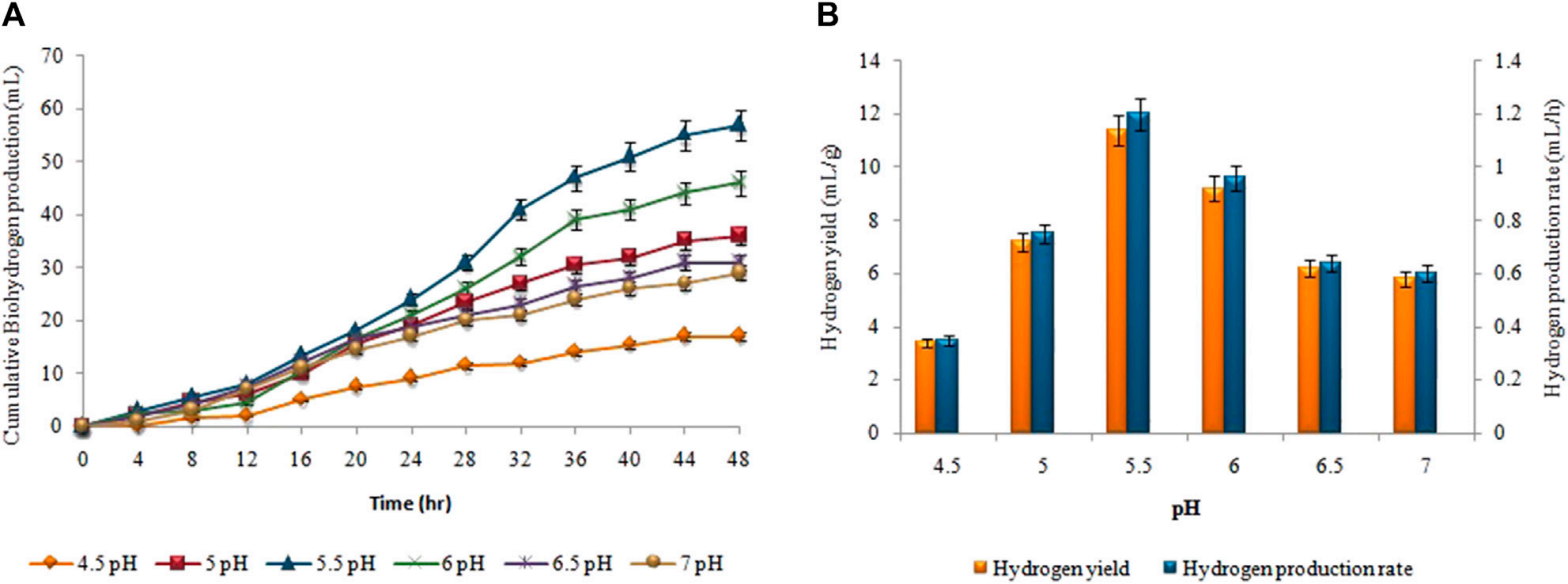
pH effect on **(A)** Cumulative H_2_ production (mL), **(B)** HY (mL/g) and HPR (mL/h).

**FIGURE 7:**
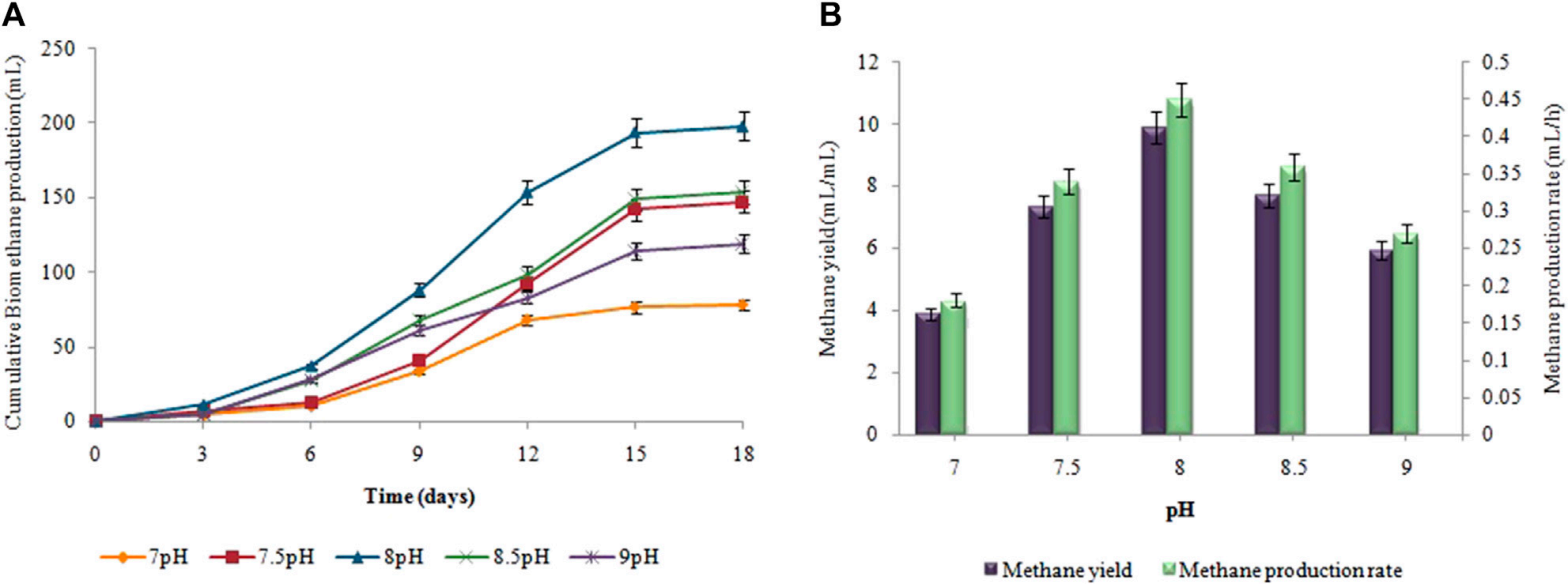
pH effect on **(A)** Cumulative CH_4_ yield (mL), **(B)** MY (mL/mL) and MPR (mL/h).

The 5.5 pH was the optimum value for achieving maximum biohydrogen production (Figure 6). At pH 5.5, the highest cumulative biohydrogen production, HY, and HPR, i.e. 57 ± 0.05 mL, 11.4 ± 0.03 mL/g, and 1.2 ± 0.01 mL/h, respectively were attained. The yield rate and production of biohydrogen steadily decreased as the pH increased. As shown in Figure 7, biomethane production steadily enhanced in all groups following a specified lag period and stopped within 18 days. The pH 8 resulted in the maximum cumulative methane production (198 ± 0.12 mL), MY (9.9 ± 0.07 mL/mL), and production rate (0.45 ± 0.05 mL/h).

### 3.5 Effect of substrate concentration on biohydrogen and biomethane production

**FIGURE 8:**
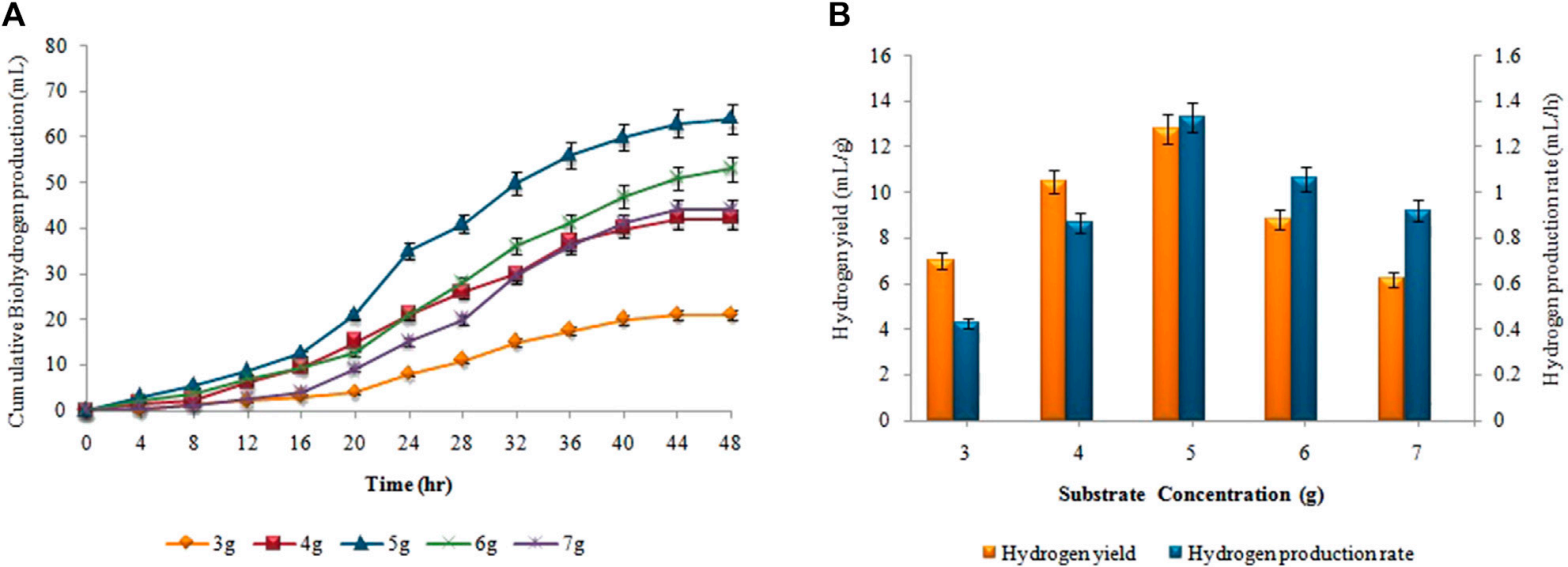
**(A)** Cumulative H_2_ production (mL), **(B)** H_2_yield rate (mL/g) and production rate (mL/h) at different substrate concentrations.

**FIGURE 9:**
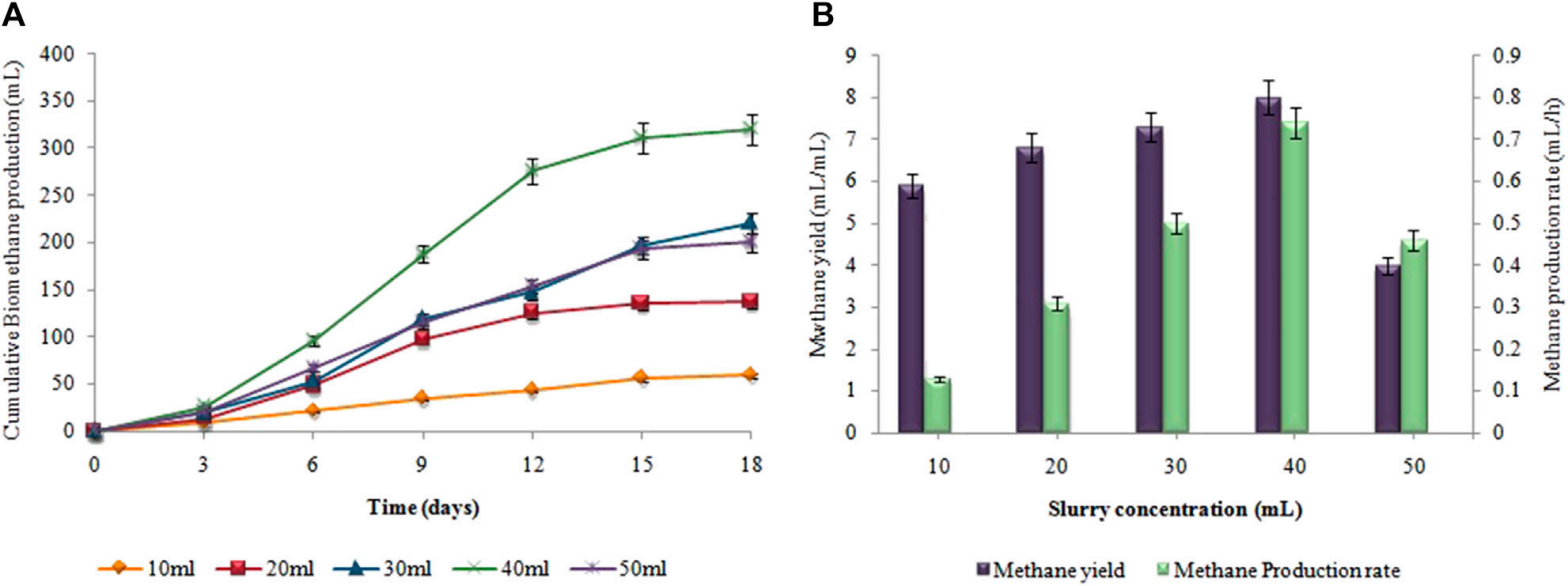
**(A)** Cumulative CH_4_ production (mL), **(B)** CH_4_ yield (mL/mL) and production rate (mL/h) at different substrate concentrations.

The supreme cumulative biohydrogen production (64 ± 0.02 mL) was achieved at 37°C after 48 h by utilizing a 5 g (w/w) substrate (Figure 8A). The maximum HY and HPR, i.e.,12.8 ± 0.01 mL/g and 1.33 ± 0.03 mL/h, respectively, were also attained by utilizing 5 g *Atriplexcrassifolia* (Figure 8B). Slurry from an enhanced dark fermentation process was used as a substrate for the synthesis of biomethane during the anaerobic digestion process. As evident in Figure 9, biomethane production steadily enhanced in all groups following a specified lag period and stopped within 18 days. The slurry/substrate concentration, i.e. 40 mL resulted in the highest cumulative methane production (320 ± 0.15 mL), MY (8 ± 0.1 mL/mL), and MPR (0.74 ± 0.07 mL/h).

### 3.6 Effect of inoculum size on biohydrogen and biomethane production

**FIGURE 10:**
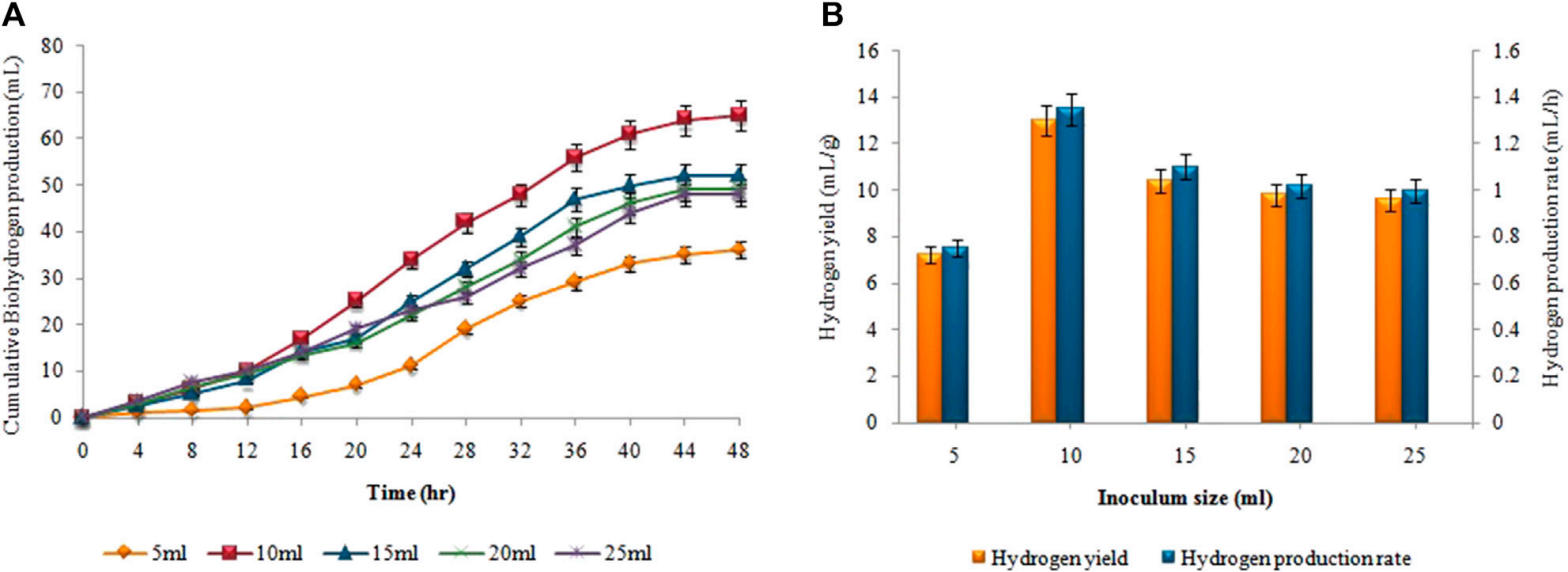
Inoculum size effect on **(A)** Cumulative H_2_ yield (mL), **(B)** HY (mL/g) and HPR (mL/h).

**FIGURE 11:**
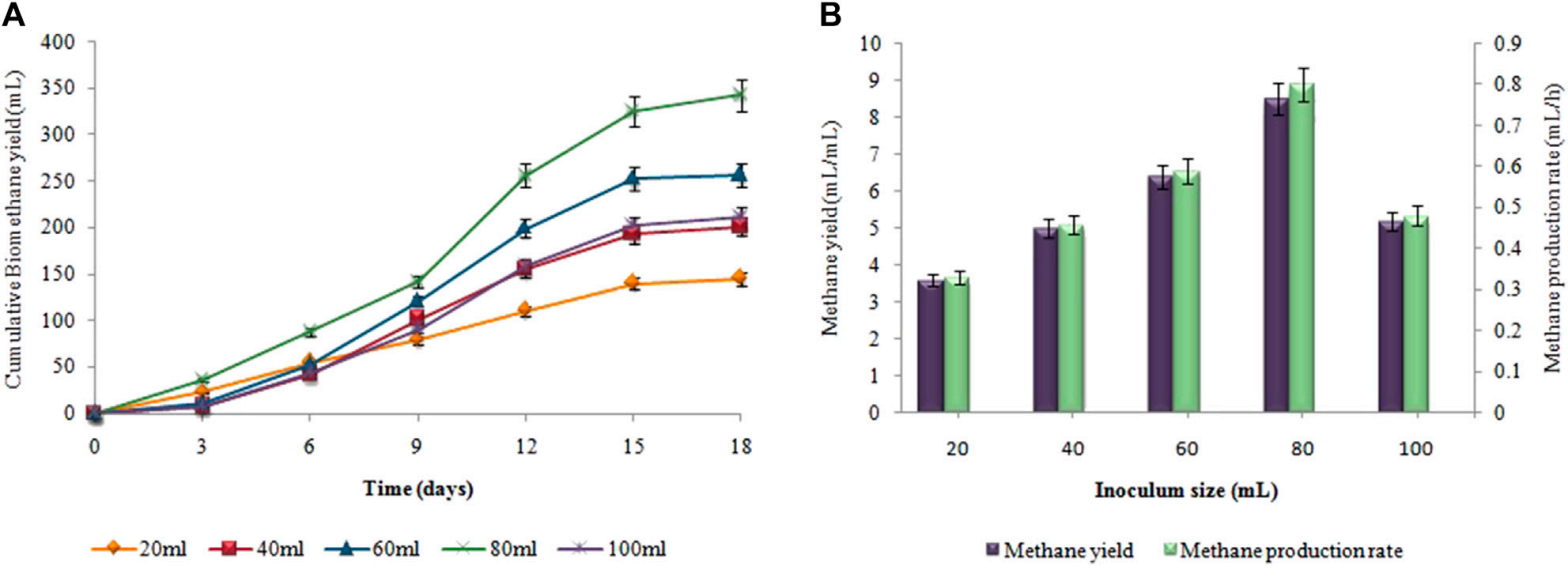
Inoculum size effect on **(A)** Cumulative CH_4_ yield (mL), **(B)** MY (mL/mL) and MPR (mL/h).

Figure 10, presents the impact of inoculum size on hydrogen yield and production rate using pretreated sewage sludge as inoculum. The maximum amount of cumulative biohydrogen, i.e. 66 ± 0.02 mL was obtained utilizing 10 mL (v/v) of sewage sludge after 48 h of incubation at 37°C (Figure 10A) According to Figure 10B, the maximal HY and HPR, i.e. 13.2 ± 0.05 mL/g and 1.37 ± 0.05 mL/h, respectively, were attained utilizing 5 g substrate and 10 mL pretreated inoculum. A continued increase in inoculum size led to a steady decline in the synthesis of biohydrogen. However, in the second stage the maximum cumulative methane production, i.e. 343 ± 0.15 mL, the highest MY, i.e. 8.5 ± 0.07 mL/mL, and the maximum MPR, i.e. 0.8 ± 0.05 mL/h were recorded after utilizing 80 mL untreated inoculum (Figure 11).

## 4 Discussion

As described earlier, a maximum delignification of 64% ± 0.01% was achieved by pretreating the *Atriplexcrassifolia* with 3% NaOH reagent. The findings were comparable to those of Chandra et al. (2012) and Zheng et al. (2018). In addition to depolymerizing lignin and providing a region for enzymatic reactions, NaOH pretreatment technique causes partial hemicellulose salvation and cellulosic deformation (Kumar et al., 2009), ultimately increasing the cellulosic content upto 62% ± 0.07%.

While talking about the effect of time on biohydrogen and biomethane generation, biomethane production significantly decreased as time increased above the optimum time limit of 18 days. This is due to the accumulation of volatile fatty acids (VFAs), increased alkalinity, and suppression of methanogenesis. Volatile fatty acids (VFAs) can suppress methane yield by inhibiting methanogenic bacteria when their concentrations become too high as time increases, disrupting the balance between acidogenic and methanogenic microorganisms. VFAs, such as butyric acid and propionic acid, can have toxic effects on methanogens, reducing methane production. Alkalinity, on the other hand is crucial for maintaining a stable pH range that supports methanogenic activity. Insufficient alkalinity can lead to a drop in pH, hindering methanogenesis and causing VFA accumulation, further suppressing methane yield. Incubation time may have a major impact on the consortium’s ability to produce hydrogen and methane since it is linked to changes in metabolic patterns and exerts an effect on the composition of subdominant microorganisms (Lu et al., 2019). The current research work’s findings support Liu et al. (2018), that the maximum biohydrogen production devoid of methane was attained after 48 h. Methanogen activity is inhibited by a shorter retention time. In contrast, the optimum incubation time for producing biohydrogen from sewage sludge, according to Massanet-Nicolau et al. (2010) was 24 h. The impact of retention time (RT) on the anaerobic digestion of lignocellulosic biomass in CSTR was studied by Shi et al. (2017), giving contrasting results. CSTRs with RT of 20 days had lower methane content than those with RT of 40 and 60 days. Propionate, predominating the reactor after a 20-day retention time, hindered the methanogens’ activity, lowering the amount of methane in the biogas. According to Zhu et al. (2008), the optimum incubation time for producing biohydrogen and biomethane can vary based on the types of substrate used, its concentration, and the design of the reactor.

The incubation temperature also significantly affects the production rates of biohydrogen and biomethane. As the temperature rose above the optimum values, i.e. 37°C and 45°C for H_2_ and CH_4_ production, respectively, the yield of both gases began to decrease, due to the inhibition of the microbial activity responsible for their production. High temperatures can negatively affect the enzymatic activity and metabolic processes of the microorganisms involved, leading to reduced efficiency and lower gas production. High temperatures impede the bioactivity of the methanogens that convert VFAs to methane (Chuang et al., 2011). It could result in the accumulation of VFAs, which would lower pH. Yokoyama et al. (2007) showed that operating a fermenter at thermophilic conditions would reduce the bioactivity of the methanogens. In the current investigation, the incubation temperature affected the production of hydrogen and methane. Temperature directly affects the thermodynamic equilibrium of biochemical pathways involved in anaerobic digestion as well as activity, growth rate, and bacterial diversification (Lin et al., 2016). A similar outcome was demonstrated by Intanoo et al. (2016), who found that cassava wastewater produced the highest cumulative hydrogen and methane at mesophilic temperatures (35°C–45°C). Contrarily, Luo et al. (2010) conducted fermentative hydrogen production utilizing cassava stillage to study the effects of temperature and pH concentrations in its enhancement. Under thermophilic temperature, the maximal hydrogen production rate, i.e. 53.8 mL H_2_/gVS, was attained. On the other hand, Zainal et al. (2020) showed how temperature and dark fermentation effluent affected biomethane synthesis in a two-stage up-flow anaerobic sludge bioreactor. Optimum biomethane production was attained at 54°C. The results demonstrated that hydrogen and methane production is dependent on temperature.

The maximum generation of biomethane and biohydrogen was achieved at pH values of 8 and 5.5, respectively. The production of both gases decreased as pH surpassed the optimized values. This can be attributed to the inhibition of the microbial consortia responsible for their production. Extremes in pH disrupt the activity and stability of the microorganisms involved, leading to reduced gas generation and lower overall efficiency. The firststage (acidogenesis) of the two-stage AD process favors the fermentation of substrates to biohydrogen and the formation of volatile fatty acids because of the operating parameters that include an acidic pH and short retention durations. The alkaline pH and extended incubation time in the secondstage promote methane production from VFAs obtained from the slurry of the firststage. The pH range in which all bacterial enzymes are active varies, with maximum activity occurring at the optimum pH value. The findings were in accordance with those reported by Lin C. Y. et al. (2011), who used mixed culture and observed maximum hydrogen productivity at an ideal pH of 5.5; Ruggeri et al. (2015) conducted a study with the objective of producing biohydrogen from wastewater from the production of noodles. The analysis of *Clostridium butyricum* revealed that a pH value of 5.5 was necessary for the highest level of hydrogen production, but a pH of 4.5 might have inhibitory effects. The maximum hydrogen yield was attained at pH = 5.5 (2.7 molH2/mol glucose) when Tapia-Venegas et al. (2015) used a pH monitoring system during biohydrogen fermentation carried out in a steady state. Won et al. (2013) observed the highest hydrogen output and productivity at a pH value of 5.5 when fermenting wastewater from a sugar refinery. The results, however, differed from those of Yang et al. (2015), who found that the highest MY from food waste was produced at pH 8.5 in contrast to the control group. Variability may occur because different pH values significantly affect enzyme activity against the substrate. VFAs reduce the pH of the reaction medium during a two-stage fermentation process, which inhibits the activity of the enzymes that produce biohydrogen and biomethane.

The production of biohydrogen and biomethane decreased as the substrate concentration exceeded the desired limit of 5 g and 40 mL, respectively. High substrate concentrations created an imbalance in microbial activity, leading to inhibition of the microorganisms responsible for biohydrogen and biomethane production, thereby reducing gas yields. In the firststage as substrate concentration increased a higher concentration of volatile fatty acids was produced. Higher substrate concentrations have been shown to increase the efficiency of hydrogen and methane synthesis, however, the maximum concentration at which product inhibition will occur is unknown. Reduced substrate concentration operates as a limiting factor and inhibits the synthesis of biohydrogen, however, if substrate exceeds its optimum limit in two-stage process, all pre-available enzymes form enzyme-substrate complexes and may not be able to bind with the residual substrate. Moreover, there is no predetermined ideal concentration for any of the substrates employed in the anaerobic fermentation process (Wang and Wan, 2009). For instance; Fan et al. (2004) stated that the best sucrose concentration for fermentative hydrogen production was 2 g/L, although earlier research showed maximum HPR at even 20 g/L (Wu et al., 2006). The use of various inoculum, substrate, and substrate concentration ranges may be the cause of this contradiction (Wang and Wan, 2009). For maximum biohydrogen production, Liu and Shen (2004) discovered that 2 g of substrate concentration produced the greatest results. Buitrón et al. (2014) discovered that 1,636 mg COD/L effluent produced the maximum results for the generation of biomethane in a two-stage process. The varied amount of VFAs and organic matter produced during the acidogenesis stage contribute to variability. The results demonstrated that hydrogen and methane production is dependent on substrate/slurry concentration.

In case of size of the inoculum, as inoculum size exceeded its optimum limit (80 mL for CH_4_ and 10 mL for H_2_), biomethane and biohydrogen production significantly decreased. The significant decrease in biomethane and biohydrogen production with an excessive inoculum size can be attributed to competition and limited availability of resources. When the inoculum size exceeds its optimum limit, there is an increased competition among microorganisms for available nutrients, leading to reduced efficiency in methane and hydrogen production. This competition can limit the growth and activity of the desired microorganisms, resulting in a significant decrease in gas production This can also be explained by the possibility that a higher inoculum size led the metabolic pathway to change, deviating from the production of biohydrogen and biomethane, towards the formation of other products. Optimum inoculum size for producing biohydrogen from pretreated sewage sludge according to Argun and Dao (2017) was 5%. Heat shock pretreatment of sewage sludge, reduces lactate synthesis, increase HY and HPR by inhibiting methanogens. According to Suksong et al. (2019) and Cremonez et al. (2019), the optimum inoculum size for producing biohydrogen and biomethane can vary based on the types of inoculum used, digestion conditions and the design of the reactor. Optimum inoculum size improves biogas disintegration, shorten the lag period, and make two-stage AD process more stable and scalable.

## 5 Conclusion

The high cellulosic content of *Atriplexcrassifolia* renders it a powerful substrate that can be utilized to produce increased yields of hydrogen and methane. Halophyte *A. crassifolia* after being pretreated with 3% sodium hydroxide yielded a significant volume of biogas when subjected to dark fermentation and anaerobic digestion. The incubation time, temperature, pH, substrate concentration, and inoculum size drastically affected the co-production of biomethane and biohydrogen. The optimal incubation time was 48 h and 18 days for biohydrogen and biomethane production, respectively. The maximum hydrogen yield and production rate were observed at an incubation temperature 37°C, substrate concentration 5 g, inoculum size 10 mL, and pH 5.5. While the maximum methane yield and production rate were obtained after incubating a reaction mixture containing 40 mL slurry concentration and 80 mL inoculum at 45°C and pH 8. Under these fully optimized conditions, a 51% and 24% increase was observed in H_2_ and CH_4_ production, respectively. This study shows that the two-stage fermentative production of H_2_ and CH_4_ from *Atriplexcrassifolia*under optimized conditions is a viable and sustainable method that can be effectively utilized at an industrial scale. Extensive exploration of the industrial potential of each component in *A. crassifolia* is needed, requiring further research to unlock valuable applications and products. Research should focus on developing cost-effective, industrial-scale pre-treatment techniques and optimizing the two-stage process for efficient and economically viable production of biohydrogen and biomethane, aligning with the increasing global demand for zero-emission fuels.

## Data Availability

The original contributions presented in the study are included in the article/supplementary material, further inquiries can be directed to the corresponding authors.
